# Whole-Cell Analysis of Low-Density Lipoprotein Uptake by Macrophages Using STEM Tomography

**DOI:** 10.1371/journal.pone.0055022

**Published:** 2013-01-31

**Authors:** Jean-Pierre Baudoin, W. Gray Jerome, Christian Kübel, Niels de Jonge

**Affiliations:** 1 Department of Molecular Physiology and Biophysics, Vanderbilt University School of Medicine, Nashville, Tennessee, United States of America; 2 Department of Pathology, Microbiology and Immunology, Vanderbilt University School of Medicine, Nashville, Tennessee, United States of America; 3 Institute of Nanotechnology (INT) and Karlsruhe Nano Micro Facility (KNMF), Karlsruhe Institute of Technology (KIT), Eggenstein-Leopoldshaffen, Germany; 4 INM – Leibniz Institute for New Materials, Saarbrücken, Germany; University of Ottawa, Canada

## Abstract

Nanoparticles of heavy materials such as gold can be used as markers in quantitative electron microscopic studies of protein distributions in cells with nanometer spatial resolution. Studying nanoparticles within the context of cells is also relevant for nanotoxicological research. Here, we report a method to quantify the locations and the number of nanoparticles, and of clusters of nanoparticles inside whole eukaryotic cells in three dimensions using scanning transmission electron microscopy (STEM) tomography. Whole-mount fixed cellular samples were prepared, avoiding sectioning or slicing. The level of membrane staining was kept much lower than is common practice in transmission electron microscopy (TEM), such that the nanoparticles could be detected throughout the entire cellular thickness. Tilt-series were recorded with a limited tilt-range of 80° thereby preventing excessive beam broadening occurring at higher tilt angles. The 3D locations of the nanoparticles were nevertheless determined with high precision using computation. The obtained information differed from that obtained with conventional TEM tomography data since the nanoparticles were highlighted while only faint contrast was obtained on the cellular material. Similar as in fluorescence microscopy, a particular set of labels can be studied. This method was applied to study the fate of sequentially up-taken low-density lipoprotein (LDL) conjugated to gold nanoparticles in macrophages. Analysis of a 3D reconstruction revealed that newly up-taken LDL-gold was delivered to lysosomes containing previously up-taken LDL-gold thereby forming onion-like clusters.

## Introduction

Current nanoscale methods to study the 3D ultrastructure of cells mostly involve TEM of thin plastic embedded sections [1], frozen sections [2], [3], edges of frozen cells [4] or fractures [5] of cellular samples. Alternatively, cells can be sectioned and imaged in a serial process [6], [7]. The spatial resolution is typically too low for protein identification in eukaryotic cells [2], [8], and specific labels consisting of heavy materials, e.g., gold, are needed to localize specific proteins in thin sections [1], [9], [10]. The nanoparticles provide contrast in electron microscopy such that the proteins can be detected in the densely packed cellular matrix. Proteins can either be sequentially conjugated to the nanoparticles and then be up-taken by living cells [11], or can be immuno-labeled with the nanoparticles in fixed plastic-embedded cells [9], [10]. The molecular machinery underlying cellular function can then be studied by localizing the individual nanoparticles, similar as is done in high-resolution fluorescence microscopy using fluorescent labels [12]. The quantitative study of nanoparticle distributions within whole cells is also of importance for the field of nanotoxicology, e.g., to study nanoparticle uptake, and for nano-medical applications [11], [13]. However, cell slicing or sectioning hinders the quantitative analysis of the 3D locations of nanoparticles at the whole cell level, because it is prone to induce artifacts including the removal of gold nanoparticles from their respective locations [11], [13].

Here, we describe a whole-cell electron microscopy method that preserves the 3D locations of gold nanoparticles conjugated to specific proteins within the cellular context, such that a quantitative analysis of the location, and the number of nanoparticles is readily possible. The staining of cellular material was kept to a minimum such to be able to image through the entire volume of the cell. STEM was used to acquire tilt series [14] of the whole-mount cells. The annular dark field (ADF) detector of STEM generates a much stronger contrast on gold than on cellular content such that gold is visible even in micrometers-thick cellular domains [15], [16]. The images mainly highlight the locations of the nanoparticles. Yet, the outlines of the cellular material are faintly visible and can be used for orientation of the location within the cell. The obtained images thus look different than what one is used to from TEM of sections containing cellular material, in which the membranes of most organelles are visible. The information contained in the images can be compared to that of fluorescence microscopy highlighting the locations of labeled proteins. The tilt range was limited to 80° (140° is common) to avoid a reduction of the spatial resolution by beam broadening through the sample at the higher tilt angles. The number of recorded images was also limited to minimize radiation damage. The 3D locations of the nanoparticles were nevertheless determined with high precision via data processing using the information that nanoparticles were present in the sample.

A not yet fully understood question in biology concerns the deleterious transformation of macrophages into foam cells during atherosclerosis [17]. It is known that LDL-derived cholesterol accumulates in macrophages, which are progressively converted into foam cells [18]. But how this accumulation occurs, i.e., what the fate is of the LDL after its intracellular trafficking remains unclear [18], [19]. In particular, it is not known whether LDL taken up at a certain time by a cell, will end up in a lysosome containing LDL that has been previously internalized. To answer the question if newly up-taken LDL is delivered to vesicles containing previously up-taken LDL, we performed STEM tomography of whole mount cells containing gold nanoparticles conjugated to LDL, and we analyzed the organization of clusters of gold nanoparticles in 3D at the nanometer scale.

## Materials and Methods

### Sample Preparation

THP-1 cells (American Type Culture Collection) were grown directly onto electron transparent silicon nitride transmission electron microscopy (TEM) support windows [20]. The THP-1 cells were differentiated into their macrophage phenotype by incubation for 4 days in RPMI media containing 10% FBS and 50 ng/ml Phorbol-12-myristate-13-acetate (PMA) [21]. Small and large gold nanoparticles were made by reduction of gold chloride using the method described by Frens [22]. Native low-density lipoprotein was conjugated to gold nanoparticles as previously described [21]. The correct preparation of the nanoparticles was verified with TEM operated at 80 keV (CM20, FEI/Philips). The diameters of the nanoparticles (n = 10) were determined in a later stage of the experiment from line-scans at the locations of nanoparticles in the tomogram of **Movie S2**, and calculating the full width at half maximum (FWHM). Incubation of the macrophages with LDL gold nanoparticles took place at 37°C in RPMI media containing 1% Fetal Bovine Serum and PMA with 8 µg/mL LDL. To prepare the samples for electron microscopy, the cells were first rinsed with Phosphate Buffer Saline (PBS), and then fixed with 1% glutaraldehyde. The cells were post-fixed with an ultra low concentration (0.001%) of osmium tetroxide [23]. Cells were then gradually dehydrated with ethanol, and critical point dried from liquid carbon dioxide [23].

### Electron Beam Stabilization of the Samples

To provide whole mount cellular samples with sufficient stability under electron beam irradiation, a layer of at least 20 nm of carbon was evaporated on the samples after critical point drying [23] (the carbon was applied using an electron beam evaporator with a base pressure of 5×10^−7^ torr and an evaporation time amounted to 45 minutes). Precautions where taken during storage and transportation of samples, including maintaining the samples under vacuum conditions to prevent rehydration. However, a certain amount of flattening of the cells may have occurred during sample preparation. This should not affect quantification, since the cell ultrastructure would be affected homogeneously. Cells were pre-irradiated during the search of the specimen for regions of interest with TEM or STEM at low magnification. The electron dose of the pre-irradiation was between 2•10^3^ to 2•10^4 ^e^−/^nm^2^, as calculated via the CM12 software [23].

### Control Experiments of LDL Gold Nanoparticles Uptake

Whole mount macrophage samples were prepared for control experiments of the uptake of LDL gold nanoparticles using TEM at 80 keV. The distribution of LDL gold nanoparticles was tested for the case of the uptake of only 14 or 5 nm-diameter nanoparticles, after one or two days of incubation. We found similar whole-cell distributions for both sizes of LDL gold nanoparticles, with a perinuclear accumulation of LDL gold nanoparticles clusters after one day of uptake. We noticed a decrease of the size of the LDL gold nanoparticles clusters along with an increase of the density of the clusters between the one and two days of incubation for both sizes of LDL gold nanoparticles. This process is likely to represent the transfer of up-taken LDL gold nanoparticles from endosomal to lysosomal compartments [24]. Furthermore, we verified that there was no effect of the size of the gold nanoparticles regarding the cellular distribution and organization of the LDL gold nanoparticles clusters after sequential uptake, by performing a first incubation with 5 nm-diameter LDL gold nanoparticles, followed by an incubation with the 14 nm-diameter LDL gold nanoparticles, i.e., the reverse order of the main experiment.

### Scanning Transmission Electron Microscopy Tomography

Scanning transmission electron microscopy (STEM) tilt series were acquired with two different microscopes. Tilt series were recorded with a Titan 80–300 ST (FEI) for a tilt range of either 76° with 2° increments, or 80° with 4° increments. The acceleration voltage was 300 KeV. The ADF detector was used. The magnification ranged between 57,000× and 115,000×. The pixel-dwell time was 4 µs. The probe current was maximal 0.1 nA. The pixel size ranged between 0.67 and 1.35 nm. The maximal electron dose that a sample was exposed to during the recording of a tilt series was 1.1•10^5 ^e^−/^nm^2^, similar as used in our previous work for the recording of a focal series [23]. The probe convergence semi-angle was 1 mrad. The image size was 2048×2048 pixels. Additional tilt series were recorded to increase the statistics of the experiments with a CM200 (FEI/Philips) for a tilt range of 70° with 5° increments. The acceleration voltage was 200 KeV, the magnification was 32,000×, the pixel size was 4.4 nm, the probe convergence semi-angle was 10 mrad, the pixel-dwell time was 10 µs, and the image size was 1024×1024 pixels. The series with the larger pixel size were used to study the locations of clusters, while the series with smaller pixel size provided information to the level of individual gold nanoparticles.

### Tomographic Reconstruction

Tilt-series were aligned using ETomo from the IMOD software package (University of Colorado, USA) [25] by cross-correlation followed by manual tracking. The LDL coated gold nanoparticles contained in the samples were used as fiducials for the alignment. The tomograms were then reconstructed using the weighted-back projection algorithm in ETomo from IMOD. The 3D models of the nanoparticles were generated with 3Dmod in IMOD. The 3Dmod slicer view was used to position the centre of each nanoparticle and to draw circles of correct diameter. Median filtering was applied to the maximal-intensity projection of a tomogram in order to erase single pixels and threshold of the intensity of the gold nanoparticles. The images were prepared for presentation with ImageJ (NIH).

## Results

### Preparation of Whole Mount Cellular Samples with LDL Gold Nanoparticles

**Figure 1:**
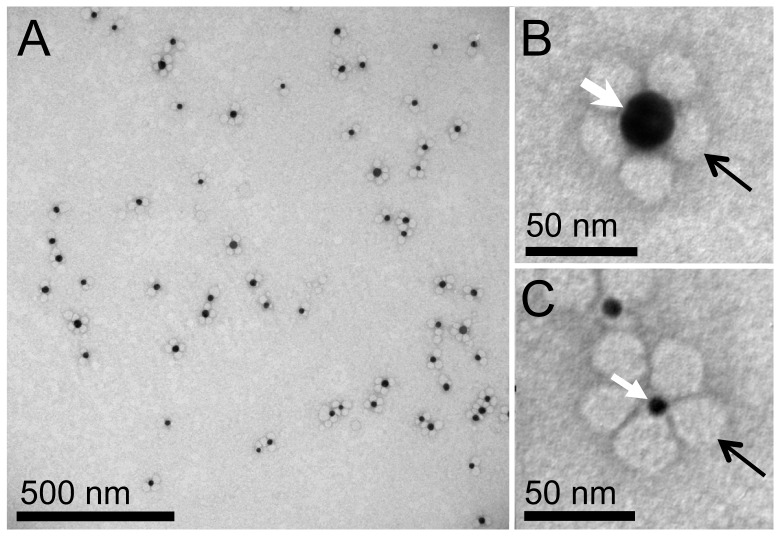
Transmission electron microscopy (TEM) of negatively stained low-density lipoprotein conjugated to gold nanoparticles (LDL gold nanoparticles). (A) LDL gold nanoparticles of 14 nm diameter (of the gold nanoparticle). (B) A gold nanoparticle (white arrow) is surrounded by low-density lipoproteins (black arrow). (C) 5 nm-diameter nanoparticle.

The uptake of native LDL was studied in whole *Phorbol-12-myristate-13-acetate*-activated THP-1 cells, a human monocyte/macrophage cell line. Macrophage cells, directly grown on thin silicone nitride TEM support membranes [23] were sequentially incubated for one day with LDL gold nanoparticles of 14 nm-diameter, and for one day with 5 nm-diameter LDL gold nanoparticles (**Figure 1**). Since cells cannot dissolve gold nanoparticles, the accumulation of gold in lysosomes serves as a marker for following the terminal fate of LDL, and the quantitation of uptake over time [21]. The cells were not stained with uranyl acetate or lead citrate but only with a low concentration of osmium tetroxide, in order to optimize the detection of the gold nanoparticles within a whole cell. As a result, the contrast obtained on membranes was lower than what is common practice in TEM of thin sections. Note that the staining level can be adjusted for a particular experiment if higher contrast on membranes is needed, but alas at the cost of the ability to image through the thicker regions of the cell. The method of critical point drying preserves the volume of the cell and fine structures such as membrane ruffles but may lead to typically 20–40% flattening of the cells [26].

The sample preparation procedure, including the coating with a thin layer of amorphous carbon, was demonstrated in a previous study to result in samples stable under electron beam irradiation during the recording of an image series [23], and we used a similar electron dose in this study. It was verified that the samples were stable under the electron beam by comparing images taken before, and after the acquisition of a tilt series. Minor changes of the cellular ultrastructure were noticed. To evaluate the effect of these changes on the analysis of the cellular compartments containing LDL gold nanoparticles, we compared the shortest and longest dimensions of seven representative clusters for pre-, and post-tilt images. The deformation of the clusters resulting from the exposure to the electron beam amounted to 1±1%, which we deemed negligible for the quantitative analysis of LDL gold nanoparticle distribution.

### Imaging a Whole Cell

**Figure 2:**
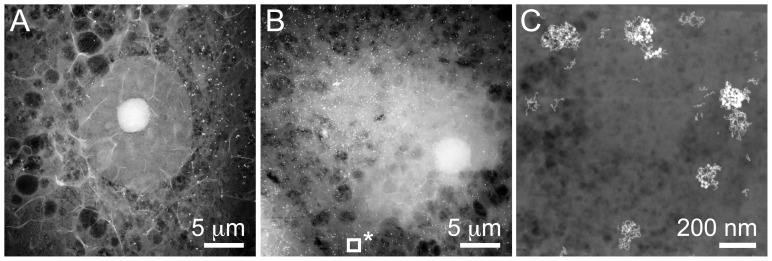
Scanning TEM (STEM) images recorded of whole-mount macrophage cells. (A) Nucleus and surrounding cellular materials. Clusters of LDL gold nanoparticles are visible as bright spots. The magnification was 5,000×. (B) Nucleus and section of cell containing many gold nanoparticles. (C) Image from a tilt-series showing gold nanoparticles of different sizes, recorded as a magnification of 115,000×. The location of this image is shown as square with * in A.

Several whole cells were imaged with STEM. A new sample was always studied with TEM at a low magnification first to find the locations of cells on the support membrane. Then STEM images were recorded at a minimum magnification of 5,000×. **Figure 2A** shows the thickest region of a macrophage cell at the position of the nucleus, observed through the SiN membrane. The nucleus appears as an oval shape (14×15 µm) at the center of the image. A nucleolus appears as a smaller and brighter round object in the middle of the nucleus; its content is apparently denser. Hole-like structures can be observed around the nucleus. These structures likely originate from lipid droplets, which are characteristic features of foam cells [18]. Finally, numerous plasma membrane ruffles can be depicted as bright and mostly straight features. On account of the low degree of staining and the use of ADF STEM, the sample was transparent for electrons even in the thickest regions. Clusters of LDL gold nanoparticles appear as bright spots. The nanoparticles concentrated in regions around the nucleus after the two days of incubation. The thickest region of a second cell is depicted in **Figure 2B**. The outline of the nucleus is less well recognizable than in the previous image, presumably on account of thicker and/or denser material surrounding the nucleus. But the nucleolus is clearly recognizable. The cell was adjacent to another cell visible in the left bottom corner. Individual LDL gold nanoparticles became visible after increasing the magnification. **Figure 2C** shows an image recorded at a magnification of 115,000×. The magnification was sufficient to distinguish between the small (5 nm) and large (14 nm) nanoparticles, and to recognize their round shapes. This image was selected from a tilt-series of which the tomographic reconstruction is available online (**Movie S1**). The location of the dataset within the cellular context is indicated by the square in **Figure 2B**. Correlation between the positions of **Figures 2B and C** was obtained by comparing the coordinates of the microscope stage used for both datasets. The whole-cell technique allows the detailed study of clusters of nanoparticles, the location of which can be selected from the overview image.

### 3D Distribution of Gold Nanoparticles within the Clusters

**Figure 3:**
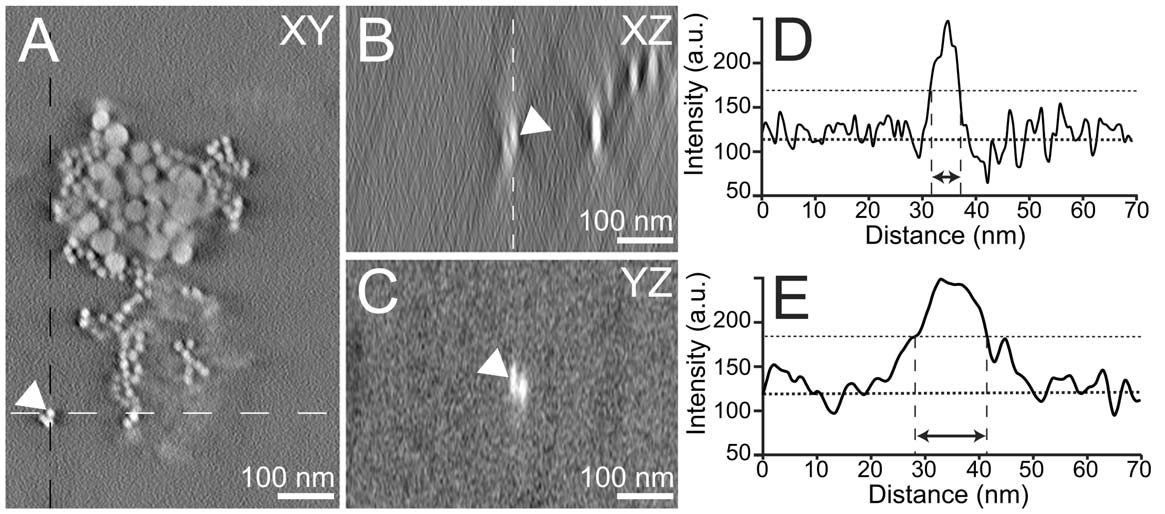
STEM tomography of nanoparticle clusters with LDL gold nanoparticles. (A) Selected region of a horizontal (xy) slice of a tomogram depicting a LDL gold nanoparticles cluster. The tilt series was recorded with a tilt range of 76° and 4° increments. The magnification was 115,000×. The pixel size was 0.67 nm. (B) Side view (xz) projection of the tomogram along the white dashed line in A. The arrow points towards the same nanoparticle depicted in A. (C) Side view (yz) projection of the tomogram along the black dashed line in A. (D) Intensity profile in xy direction of the nanoparticle depicted in (A). Intensity is plotted along the white dashed line in A. (E) Intensity profile in xz direction of the nanoparticle depicted in A. The intensity is plotted along the white dashed line in B.

Up-taken proteins are transported in cells to be ultimately degradated in lysosomes [19]. Because gold nanoparticles cannot be degraded by lytic enzymes, they can be used as fiducials to follow the fate of proteins during their intracellular trafficking [21]. We studied the fate of newly up-taken LDL by analyzing the 3D spatial distribution of the individual gold nanoparticles *within* intracellular clusters. **Figure 3A** shows a selected region of a horizontal slice of a tomogram, depicting a cluster of gold nanoparticles (**Movie S1**). Both sizes of nanoparticles can be recognized. The nanoparticles appear oval instead of round in the side views of a section through the same cluster (**Figures 3B, C**), which is a consequence of the limited tilt angle range of the tilt series. The respective intensity profiles (line scans) over a nanoparticle (arrow in **Figures 3A, B**) were analyzed (**Figures 3D, E**). The full width at half maximum (FWHM) of the intensity peaks in the horizontal-, and vertical direction amounted to 6 and 13 nm, respectively. The measured dimension in horizontal direction reflects the actual size of the nanoparticle and the horizontal resolution is thus 6 nm or better. Since the nanoparticles were spherical, it can be concluded that the point spread function in vertical direction was much larger than the size of the nanoparticle. In case the point spread function is much larger than the object under observation, this object can be considered as point object, and the FWHM serves as measure of the resolution. The resolution in vertical direction thus amounted to 13 nm. To calculate the theoretical z-elongation factor (e_yz_) of the nanoparticles in the tomograms, we used an equation derived from the *Crowther Criterion*, that calculates the degradation of the information in z axis due to the missing wedges in tomogram reconstructions [**27**]:
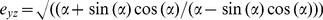
(1)with α maximal tilt angle, in radians, measured between the 0° tilt and the absolute maximal tilt. In our STEM tomography experiments, we used tilt angle ranges of 70°, 76° and 84°. According to equation (1), the corresponding expected z-elongation factors are equal to 2.77, 2.54 and 2.28, respectively. This amount of vertical elongation is consistent with the experimental value for the used tilt angle range.

### Determining the Locations of the Nanoparticles

**Figure 4:**
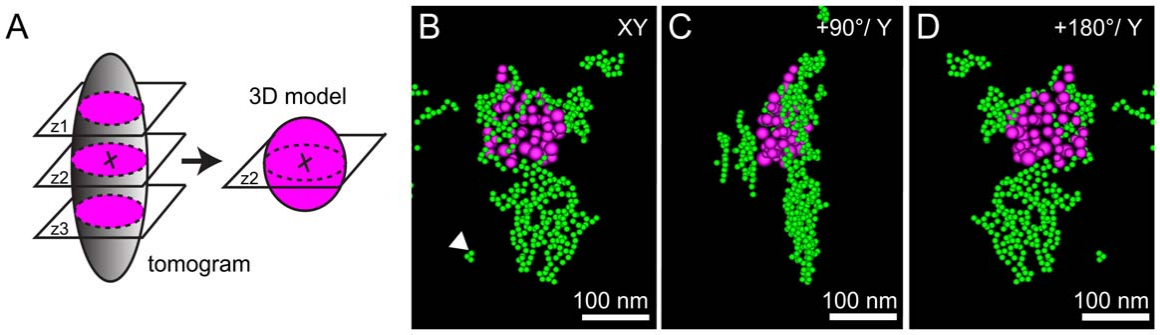
Generating a 3D model of a nanoparticle cluster. (A) Schematics explaining the construction a 3D model of a nanoparticle. (B) 3D model of LDL gold nanoparticles cluster shown in (A) viewed along the y-axis, (C) rotated +90°, and (D) +180°. Scale bars, 100 nm.

Including known information about the sample geometry in the calculation of the 3D reconstruction may lead to a model of high precision despite a limited set of tilts [28]. The vertical locations of the nanoparticles in our samples were determined with a higher precision than the vertical resolution by using the information that the nanoparticles were of spherical shape, and that most of them were sufficiently separated in space such that each individual nanoparticle could be distinguished. A 3D model of each nanoparticle was generated from its 3D location, and diameter (**Figure 4A**). The 3D dataset of the tomogram of Figure 3C (**Movie S2)** was first searched for the vertical position at which a particular nanoparticle was in focus (the slice with maximal diameter was selected). The horizontal location (the x and y coordinates of the center of the nanoparticle), and the diameter of this nanoparticle were then determined from this slice. A sphere with corresponding diameter was finally positioned at the same 3D location in a newly defined dataset. This procedure was repeated for all nanoparticles contained in the clusters of selected perinuclear regions. The 3D model of **Movie S2** contained four clusters with an average of 27±11 large, and 230±39 small nanoparticles per cluster. A total of 321 remaining nanoparticles were located in clusters containing small nanoparticles only. A total of 1355 (115 large and 1240 small) nanoparticles was analyzed for this dataset.

The precision at which the nanoparticles were located was on average ±0.3 and ±2 nm for the horizontal and vertical direction, respectively. In order to determine the vertical precision, we analyzed 26 small nanoparticles, and searched in which vertical plane(s) each nanoparticle appeared in focus. Indeed the nanoparticles presented a maximum diameter over several consecutive planes (generally 3–4), and thus appeared in focus over consecutive vertical planes. We consistently selected as focus plane the median plane inside this vertical range. For each nanoparticle, we thus obtained a number of planes in which these nanoparticles were in focus, a vertical focus range, and we calculated the standard deviation of these values for the 26 nanoparticles analyzed as a measure of the vertical precision.

For horizontal precision, after the vertical focus planes of 20 small nanoparticles were selected, we measured the xy coordinates of the geometric center of these nanoparticles in the horizontal direction. The geometric center was visually selected. For each nanoparticle, we repeated 5 times the positioning and measurements of the coordinates of this geometric center. We calculated the standard deviation of these coordinates. We used the calculation of the mean value of these standard deviations to deduce the error in horizontal positioning. The analyzed nanoparticles were selected from different vertical positions.

### Classification of Gold Nanoparticle Clusters

**Figure 5:**
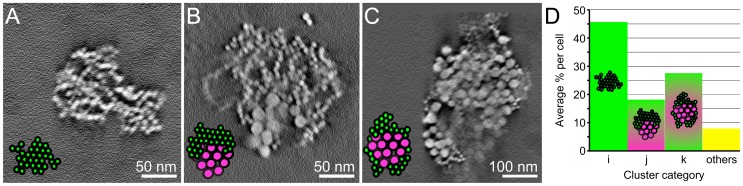
Quantitative characterization of the three main types of clusters formed after sequential incubation with large, and small LDL gold nanoparticles. (A–C) Representative images of the three main categories of clusters. (D) Population of each cluster type in an average thickness of 0.73±0.29 µm. Scale bars, 100 nm.

The 3D reconstructions revealed that gold nanoparticles from the first day of incubation (14 nm-diameter) were surrounded by those of the second incubation day (5 nm-diameter) for many clusters. **Figures 5B–C** show three projections of such a cluster (**Movie S3**), selected from the dataset shown in **Movie S2**. On account of the 3D model it is possible to conclusively tell that the cluster has a shell of nanoparticles of the second day around those of the first day, which cannot be derived from two-dimensional data. The cluster as shown in **Figures 5B–C** preserved all its cargo throughout sample preparation since sectioning was avoided. Even though the 3D data involves a whole mount cell, the locations of the gold nanoparticles were determined with a precision of a magnitude smaller than the diameters of the nanoparticles, thus allowing a precise analysis of the clusters at the level of individual nanoparticles. Several different types of clusters were found. The occurrence of different types of clusters was classified from a total of 84 clusters from five tomograms. The main types found were, i-type clusters, containing only second day (small) nanoparticles (**Figure 5A**), j-type clusters, with groups of both types of nanoparticles adjacent to each other (**Figure 5B**), k-type clusters, in which the first day gold nanoparticles were surrounded by second day gold nanoparticles (**Figure 5C**), and other types of clusters containing only first up-taken nanoparticles, or consisting of a spatial mixture of small, and large nanoparticles. Quantification revealed that about half of the gold nanoparticles clusters were of i-type, while the j- and k-types represented about a quarter of the population each (**Figure 5D**).

### Distribution of LDL Gold Nanoparticles Clusters throughout the Cell

**Figure 6:**
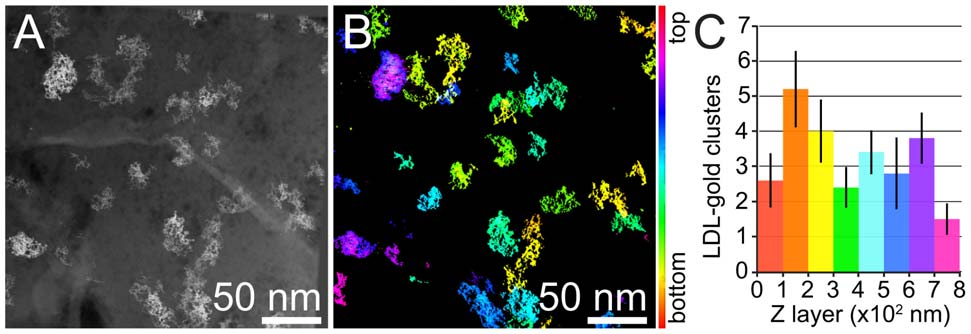
STEM tomography of a whole macrophage after sequential incubation with large (14 nm diameter), and small (5 nm) LDL gold nanoparticles. (A) 0° STEM view through the thickness of a whole-mount macrophage. The magnification was 57,000×. The pixel size was 1.35 nm. (B) Tomogram reconstructed from an 80° tilt series with 2° increments containing the 0° view in A. The vertical position of the LDL gold nanoparticles clusters is color-coded. (C) Quantification of the vertical positions of the LDL gold nanoparticles clusters within 0.9±0.1 µm thick perinuclear regions. The mean number of clusters per 100 nm-thick virtual cell region (color bars), and standard deviations (black bars) are represented.

Finally, we asked whether clusters of nanoparticles would exist throughout the entire cellular thickness or whether preferential vertical regions would exist. **Figure 6A** shows a 0°-tilt STEM view of a 0.79 µm thick region, with LDL gold nanoparticles clusters located at different vertical positions in the cell. The vertical position of each cluster was determined with respect to the bottom of the cell adhering to the SiN window. The vertical position of the lowest nanoparticle appearing in the tomogram was used as a reference of the bottom, since this nanoparticle consistently coincided with the appearance of the intracellular space. **Figure 6B** shows the color-coded vertical position of the clusters in the tomogram corresponding to **Figure 6A**.

In order to study the vertical distribution of the clusters throughout cells, the data of five tilt series acquired in 0.9±0.1 µm thick perinuclear regions were analyzed, including a total of 127 clusters. A cluster was considered as such if it contained at least 10 nanoparticles since the number of single nanoparticles (far separated from others) and of clusters less than 10 nanoparticles were negligible. The datasets were divided in vertical regions of 100 nm thickness, and the number of clusters in each vertical region was counted. As can be seen in resulting statistics of **Figure 6C**, the gold nanoparticles clusters distributed throughout a vertical range of 0.8 µm. This value is likely to correspond to the thickness of the cell body in the regions we analyzed, since nanoparticles distributed in the intracellular space below the top plasma membrane protrusion. A maximal thickness of 1.5 µm was measured from the bottom of a cell towards its most upward protrusions in the perinuclear region. The thickness of the cells is comparable to other thin cells flattened on a silicon nitride support membrane [29]. The resulting statistics of **Figure 6C** shows that the clusters were found throughout the thickness of the main cell body with no preferential vertical position for the LDL gold nanoparticles. This experiment provides an example of a quantitative analysis possible with a whole cell sample.

## Discussion

Proteins taken up by cells are known to be internalized by endosomes (endocytosis), and then to be ultimately delivered to lysosomes, the terminal cellular compartments that degrade proteins [19]. We assumed that the observed clusters of nanoparticle represent endosomes or lysosomes, although their membrane structures were not visible on account of the low concentration of staining as needed to be able to image through the entire cell. But it has been previously demonstrated that LDL-gold nanoparticles taken up by macrophages follow the endosomal lysosomal degradative pathway, with electron microscopic studies of thin sections showing LDL-gold nanoparticles accumulations in lysosomes [21], [30]. We speculate that the targeting of LDL-gold conjugates to lysosomes is not specific to LDL and macrophages but that it rather appears as a general trafficking mechanism of gold-conjugated proteins, as for example occurs with bovine serum albumin serum (BSA)-gold conjugates which accumulate in the lysosomes of normal rat kidney cells [24].

**Figure 7:**
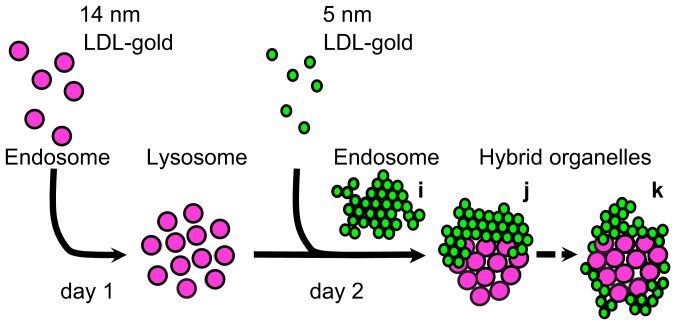
Interpretative scheme of the mechanism that causes the different categories of LDL gold nanoparticles clusters.

Our analysis of the 3D organization of the gold nanoparticle clusters formed after sequential uptake of LDL gold nanoparticle conjugates revealed that the gold nanoparticles of the second day associated within clusters of gold nanoparticles of the first day. These results are consistent with the literature [21], [30]. Based on our observations and quantifications of intact gold nanoparticle clusters, we propose that the three main types of observed gold nanoparticle clusters (i, j, and k) reflect successive steps of the compartmental trafficking of LDL (**Figure 7**): LDL gold nanoparticles of the second day of incubation are likely collected in endosomes (i-type cluster), and those are delivered to lysosomes [24] filled with the gold nanoparticles of the first day (j-type clusters). This view is supported by our finding that clusters containing only first-day gold were present in a minor fraction at the second day. We thus formulate the hypothesis that the surrounding of the first day gold by that of the second day (k-type clusters) represents the final step of LDL’s intra-cellular trafficking within the observation-time window. In this model, newly up-taken LDL is delivered to lysosomes containing LDL of a previous incubation day, giving rise to a shelled (onion-like) 3D organization of the nanoparticles. The occurrence of this shelled organization could possibly be a consequence of the formation of multi-lamellar lysosomes. Indeed, multi-lamellar lysosomes are present in macrophages foam cells and contain layered membranes [30], [31]. We formulate the hypothesis that in such compartments, the endosomal and lysosomal membranes could be layered after fusion of these two entities [19], [32], and would be responsible for the shelled organization of the gold nanoparticles. Our hypothesis may have relevance for the disease atherosclerosis in pointing at such compartments [33].

In our additional experiments (**Figure 6**) we found that on average the LDL gold nanoparticles clusters were found distributed in the cell bodies with no preferential vertical position. These findings suggest that the lysosomes used for LDL degradation do not accumulate at preferential cellular levels, but rather distribute homogeneously in the cytoplasm along the height of the cells.

The 3D locations of the smaller (5 nm-diameter) gold nanoparticles conjugated to LDL were determined throughout the entire volume of a macrophage cell. STEM tomography is already capable of imaging sections of about a micrometer in thickness [14], [34]–[36], much thicker than what is possible with the same resolution with TEM. But the quantification of nanoparticles in sections can only be conducted relative to the analyzed thickness, and one does not know how many nanoparticles are lost cutting the section. Moreover, it is experimentally challenging to cut sections parallel to a defined plane with respect to the cellular body. The whole mount cell approach we used in this study avoided these sectioning-related risks of artifacts, and complications for quantification [11], [13]. In our analysis we included all nanoparticles in the cellular volume. When analyzing the 3D shapes of the clusters on nanoparticles we thus always knew that each cluster represented its entire volume, and was not cut through. Information of a whole cell can be reconstructed from stitched tomograms recorded on sections but this method involves a highly time-consuming procedure [37], and still removes a fraction of the material and thus nanoparticles at the locations of the cuts. The whole mount technique in combination with labels is a much faster method for whole cell analysis compared to whole cell analysis via the stitching of sections for studying the locations of a set of labels. In this initial study we used manual detection of the nanoparticle but detection can readily be automated, for example, by 3D by applying the missing wedge to a spherical phantom particle and searching the tomogram for similar objects using a cost function like cross correlation. A further advantage is the easy at which it is possible to orient within the 3D volume of the cell using the low magnification STEM images.

The STEM images highlight the locations of labeled proteins while the contrast on the organelle membranes is rather low. This type of image is uncommon in electron microscopy, since one is used to see TEM images showing the ultrastructure of cells. An analogy can be found in light microscopy, where fluorescence microscopy of labeled proteins is used for achieving biological insight, since the past decade with sub-diffraction resolution [12], [38]. Note that fluorescence microscopy of course requires much less sample preparation. The labels usable for STEM are nanoparticles of heavy materials of various sizes and shapes. The surface of the nanoparticles can be functionalized to prevent unspecific interactions with the proteins of the cell medium serum [39]. A variety of techniques is available to introduce nanoparticles into cells [40]: i) specific or unspecific physiological cellular uptake by endocytosis [41]; ii) facilitated delivery by conjugating the nanoparticles to cell-penetrating peptides [42], liposomes [43], transfection reagents [44] or bacterial toxins [40]; or iii) active delivery using physical methods that disrupt the cell membrane (sonoporation [45], microinjection [46], gene-gun [47]). The nanoparticles can be fluorescent, such as Quantum Dots [48], [49], and enable correlative light-electron microscopy studies [50]. New methods are also available to avoid the introduction of nanoparticles into cells, by genetically labeling intra cellular proteins for electron microscopy [1], [51]–[53].

### Conclusions

This work shows that STEM tomography of whole mount cellular samples can be used to analyze the 3D spatial distribution of gold nanoparticles conjugated to proteins within the context of a whole cell. The precision was sufficient to determine the 3D locations of LDL gold nanoparticles of a diameter of 5 nm. 3D reconstructions were generated of clusters containing LDL gold nanoparticles of two different sizes. The results indicate that newly up-taken LDL is delivered to lysosomes containing LDL of a previous incubation day. The same method may proof useful for nanoparticle uptake studies in the context of research on nanotoxicology, and molecular imaging. Our technique avoids sectioning, which is prone to induce artifacts hindering quantitative analysis of nanoparticle locations. However, the disadvantage is a low contrast on the biological material. Similar as in fluorescence microscopy our method can be used to study a selected set of labels. We anticipate that STEM tomography in combination with nanoparticle labeling will be broadly applied to explore the 3D locations of various types of proteins in whole cells, and to the quantification of nanoparticle uptake.

## Supporting Information

Movie S1 **Scanning transmission electron microscopy (STEM) tilt series of a macrophage sequentially incubated with large, and small (14 and 5 nm, respectively) LDL-gold.** The tilt series was recorded for a tilt range of 76° with 4° increments.
(MOV)

Movie S2 **3D modelling of the nanoparticles shown in Movie S1.** Four clusters contained on average 27±11 large (magenta) and 230±39 small (green) nanoparticles per cluster. 321 remaining nanoparticles were located in clusters containing small nanoparticles only. Scale bar 200nm.
(MOV)

Movie S3 **Selected region of the 3D model of Movie S2 highlighting one cluster.** A total of 293 small nanoparticles surrounded 57 large ones. Scale bar 200 nm.
(MOV)
